# The kent meningococcal outbreak 2026: a wake-up call for antimicrobial stewardship, vaccine policy and outbreak preparedness

**DOI:** 10.1093/jacamr/dlag066

**Published:** 2026-04-27

**Authors:** Rasha Abdelsalam-Elshenawy

**Affiliations:** AMR/AMS Global Health Policy Expert, School of Health, Medicine and Life Sciences, University of Hertfordshire, Hatfield, UK

## Abstract

The 2026 Kent meningococcal outbreak, involving 20 confirmed or suspected cases and two deaths within days, rapidly escalated from a local cluster into a coordinated national prescribing response within 72 hours. This response demonstrated timely public health action, characterized by the rapid deployment of chemoprophylaxis, clear communication, and coordinated delivery across UKHSA, NHS England, and general practice. At the same time, it highlighted opportunities to further strengthen structured, multi-sector collaboration in outbreak management. The event highlights the importance of aligning antimicrobial stewardship (AMS), immunization policy, and outbreak preparedness systems. The expansion of chemoprophylaxis through general practice enabled rapid and equitable access to antibiotics, while reinforcing the need for risk-stratified assessment frameworks to support targeted antibiotic use at scale. With MenB identified as the predominant strain, the outbreak also exposes a structural immunization gap among adolescents and young adults who fall outside the routine UK childhood vaccination programme, thereby increasing reliance on antibiotic-based prevention strategies. Drawing on the GUIDE Framework, this Viewpoint advocates for embedding AMS metrics into outbreak response, strengthening MenB immunization strategies, and operationalizing risk-stratified prophylaxis to enhance system resilience and ensure more sustainable, evidence-based outbreak management.

## Introduction

In March 2026, the United Kingdom experienced one of its most significant outbreaks of invasive meningococcal disease (IMD) in recent years. Centred in Canterbury, Kent, and linked to a university nightclub, the outbreak resulted in at least 20 confirmed or suspected cases and two deaths within days.^1^ Within 72 hours, the response escalated nationally: on 18 March 2026, UKHSA and NHS England instructed general practitioners to provide chemoprophylaxis to returning contacts on request.^2^

This rapid shift from localized prescribing to nationwide prescribing highlights tensions among antimicrobial stewardship (AMS), immunization policy, and outbreak preparedness. The GUIDE Framework (Gather Evidence, Urgency and Source, Identify Choice, Define Duration, Evaluate and Ensure Review) supports proportionate, patient-centred prescribing aligned with WHO AWaRe and Start Smart Then Focus.^3^ Applied to IMD contact management, it structures risk assessment, prophylaxis selection, duration, and review.^3^ The Kent outbreak, therefore, represents both a public health emergency and a policy-defining moment. This Viewpoint examines these intersections through the GUIDE Framework to identify key AMS gaps and inform more balanced outbreak responses.^3^

## Clinical context: why speed defines outcome

Meningococcal disease can present as meningitis, septicaemia, or both, with rapid deterioration possible within hours.^1^ Early symptoms, including fever, severe headache, vomiting, myalgia, and cold extremities, are non-specific and may be mistaken for influenza or, in student populations, a hangover.^1^ A non-blanching petechial or purpuric rash may occur and can be assessed using the glass test; however, this is often a late sign and may be absent.^4^ On darker skin tones, the rash may be visible only on the palms, soles, roof of the mouth, or inner eyelids.^4^ Prompt recognition and urgent response are essential. NICE guideline NG240 recommends immediate referral to secondary care via emergency services for suspected IMD; in the UK, this means calling 999. The guidance emphasizes that hospital conveyance must not be delayed to administer antibiotics; however, parenteral benzylpenicillin or ceftriaxone may be administered in primary care settings if significant delays in transfer occur.^5,6^ Vaccination remains the cornerstone of prevention in the UK. The NHS MenACWY programme, introduced in 2015, offers a single dose to teenagers in school year 9/10 and remains available as a catch-up for eligible individuals until their 25th birthday, including university entrants. It provides protection against serogroups A, C, W, and Y; however, national adolescent coverage remains below the 95% target threshold.^7^ Notably, MenB accounted for 87% of all confirmed IMD cases in the UK in 2021–2022, yet MenB vaccines are not part of the adult programme, creating a critical prevention gap that drives reliance on antibiotics.^7,8^

## National meningococcal chemoprophylaxis: prescribing and antimicrobial stewardship

UKHSA’s rapid deployment of over 2500 prophylactic antibiotic doses to students, close contacts, and nightclub visitors was clinically justified and aligned with established meningococcal contact management guidance.^9^ The UK guidance defines ‘close contact’ for meningococcal prophylaxis as household contacts or individuals with equivalent levels of exposure, such as prolonged face-to-face contact (typically greater than 8 hours) or direct exposure to oral secretions.^9^ The broader offer to nightclub attendees served as a precautionary public health measure, reflecting the practical difficulty of precisely identifying exposure levels in crowded, high-risk settings where contact tracing is inherently imprecise. Ciprofloxacin and rifampicin remain central to secondary prevention, effectively interrupting carriage and transmission among high-risk contacts. The subsequent national escalation, through a joint UKHSA/NHS England letter to Integrated Care Boards, enabled general practitioners to provide chemoprophylaxis to returning contacts on request, ensuring timely and equitable access for students dispersed across England.^2^ This reflects a responsive and patient-centred public health approach, extending protection through primary care beyond traditional contact tracing pathways. It is important to acknowledge, however, that antibiotic prophylaxis in younger adults is not without risk. Potential adverse effects include gastrointestinal disturbances, photosensitivity reactions associated with ciprofloxacin, and the population-level concern of promoting antimicrobial resistance (AMR). These considerations further emphasize the need for risk-stratified approaches and reinforce vaccination as the optimal long-term prevention strategy. Importantly, this approach creates an opportunity to further strengthen AMS. Integrating real-time surveillance of prescribing patterns, adherence, and resistance signals would enhance understanding of population-level impact while safeguarding long-term antibiotic effectiveness. The guidance also appropriately reinforced that hospital conveyance must not be delayed for antibiotic administration, emphasizing that antibiotics are a supportive measure within a broader clinical pathway.^2^ Embedding prospective AMS surveillance within outbreak frameworks would be a valuable next step.

## The vaccine gap: when prevention fails, antibiotics fill the void

The Kent outbreak has highlighted a well-recognized but insufficiently addressed immunization gap. MenB, confirmed as the causative serogroup in multiple cases, remains the leading cause of IMD in the UK.^1^ Although routine infant MenB vaccination was introduced in 2015, individuals born before May 2015, including most university students, are not covered.^5^ The MenACWY programme offers no protection against MenB, and the two licensed MenB vaccines available in the UK, Bexsero^®^ (GSK) and Trumenba^®^ (Pfizer), are largely accessed privately, limiting uptake among young adults.^6^

From an AMS perspective, this gap represents a clear opportunity. Incomplete vaccine coverage allows preventable infections to occur, increasing reliance on antibiotic prophylaxis during outbreaks. The Kent response illustrates this, with large-scale chemoprophylaxis and national prescribing guidance implemented in an under-protected population. Strengthening MenB immunization coverage would reduce antibiotic reliance while improving population protection. Recent commitments to review eligibility criteria signal positive policy momentum.^6^ Campaigns such as Meningitis Now’s *No Plan B for MenB*, advocating expanded access and uptake, provide a constructive framework to advance both public health and AMS objectives.^7,8^

## Outbreak response through an AMS policy lens

The Kent outbreak response has been coordinated, transparent, and appropriately scaled, demonstrating strong public health leadership. From an AMS perspective, it also highlights opportunities to further strengthen outbreak preparedness.

First, developing risk-stratified chemoprophylaxis protocols would support a shift from broad, demand-led prescribing towards targeted, exposure-based interventions. Pre-agreed frameworks with clear eligibility thresholds, prescribing pathways, and monitoring requirements could enable more proportionate antibiotic use while maintaining effective public protection. Second, improved integration between outbreak-related prescribing data and national AMR surveillance systems would enhance visibility of antibiotic use during incidents. Linking these data streams would allow timely identification of prescribing patterns and resistance signals. These ambitions align with the UKHSA national action plan, IDSA/SHEA core stewardship principle of preauthorization and prospective audit and feedback, and the UK's Start Smart Then Focus (SSTF) ‘Then Focus’ review cycle.^9,10^ Applying the GUIDE Framework (Gather, Urgency, Identify, Define, Evaluate) to IMD contact management operationalizes these principles across all five prescribing decision points, from risk assessment to post-prophylaxis review (Supplement S1, available as Supplementary data at JAC-AMR Online), which calls for optimizing antibiotic prescribing and strengthening AMR surveillance systems across all sectors, including outbreak settings.

Second, outbreak-related prescribing data must be integrated in real time with national AMR surveillance systems. This directly supports the UK NAP for AMR 2024–2029 commitment to optimize antibiotic use and strengthen surveillance across all sectors, and aligns with WHO AWaRe monitoring targets, which set a threshold of at least 60% of total antibiotic consumption comprising Access-group antibiotics, a threshold that large-scale fluoroquinolone deployment during outbreaks risks undermining.^11^

Third, outbreak preparedness must encompass antibiotic supply chain planning. The Kent response required rapid deployment of over 2500 antibiotic doses across primary care, highlighting a gap in pre-positioned antibiotic stock management for outbreak scenarios. Ensuring adequate, rapidly deployable antibiotic supplies, coordinated through NHS England and integrated care boards, is an essential but currently pivotal component of national outbreak readiness.

**Figure 1.**
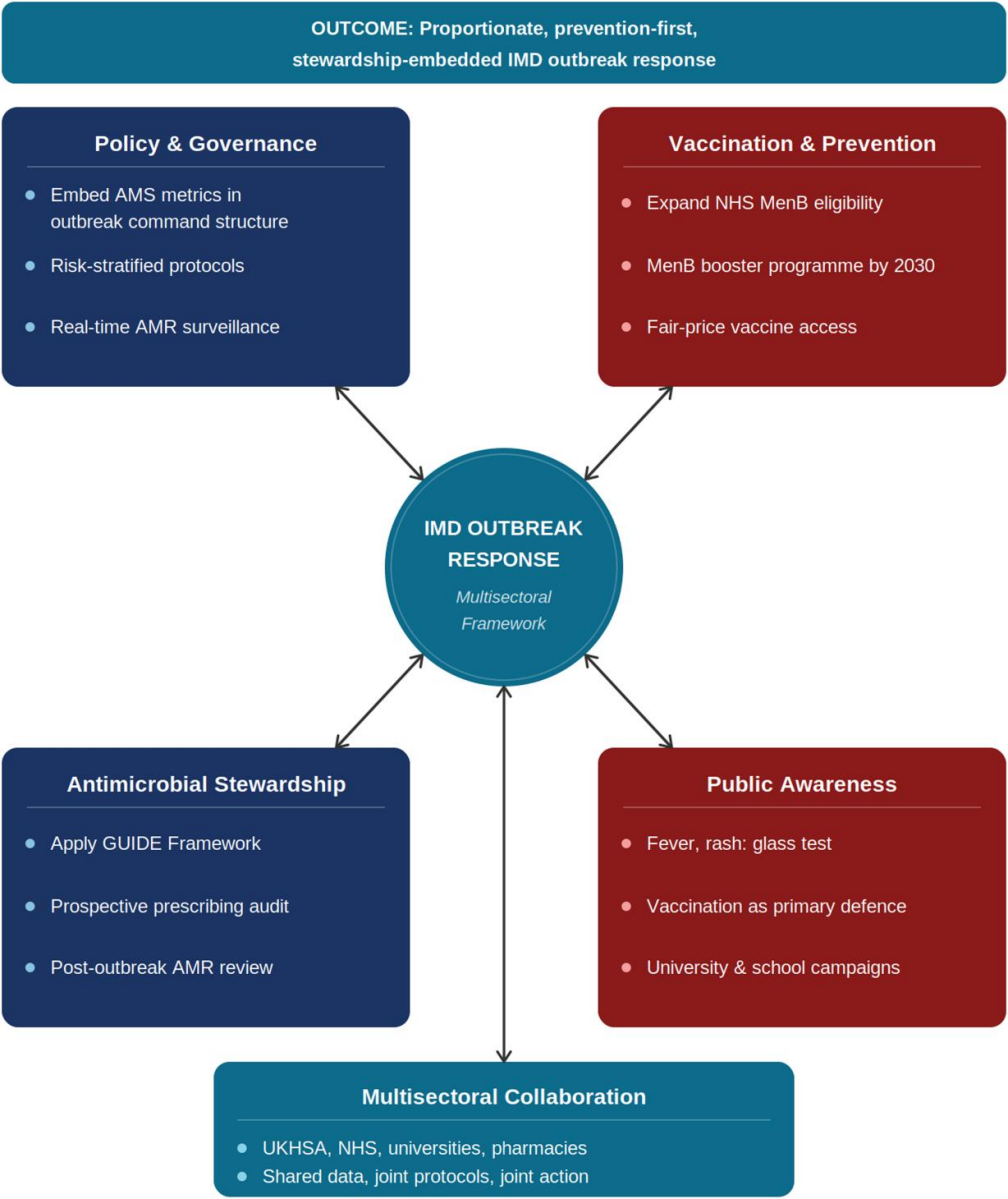
Multisectoral framework for a proportionate, prevention-first, stewardship-embedded invasive meningococcal disease outbreak response. Five interdependent domains are required for an effective IMD outbreak response: Policy and Governance (embed AMS metrics, pre-agreed risk-stratified protocols, real-time AMR surveillance); Vaccination and Prevention (expand NHS MenB eligibility, MenB booster programme, fair-price access); Antimicrobial Stewardship (apply the GUIDE Framework: Gather, Urgency, Identify, Define, Evaluate); Public Awareness (symptom recognition, vaccination as primary defence, community campaigns); and Multisectoral Collaboration (UKHSA, NHS, RPS, universities and public health charities). Bidirectional arrows indicate interdependencies between domains. AMS = antimicrobial stewardship; AMR = antimicrobial resistance; GUIDE = Gather Evidence, Urgency and Source, Identify Choice, Define Duration, Evaluate and Ensure Review; IMD = invasive meningococcal disease; MenB = meningococcal group B; NHS = National Health Service; RPS = Royal Pharmaceutical Society; UKHSA = UK Health Security Agency.

Fourth, communication must reinforce that vaccination is the only durable prevention and that rapid hospital assessment takes priority over prophylaxis in IMD management. Figure 1 illustrates how these domains align within a stewardship-embedded, prevention-first outbreak response.^3^ The GUIDE Framework structures IMD contact management across five steps, highlighting areas for strengthened AMS integration (Supplement S1). Figure 1 illustrates how these domains align to support a prevention-focused, stewardship-embedded outbreak response.^3^

## Conclusions and policy recommendations

The 2026 Kent meningococcal outbreak represents both a public health tragedy and a policy opportunity. Its rapid escalation highlights how IMD can outpace locally calibrated AMS systems and highlights the need for integrated responses. Four reforms are proposed: embedding AMS metrics within outbreak management to enable real-time monitoring of antibiotic use and resistance; closing the MenB vaccination gap by expanding eligibility to adolescents and young adults, reducing population-level antibiotic dependency; developing risk-stratified chemoprophylaxis protocols to support targeted, proportionate prescribing; and strengthening antibiotic supply chain preparedness by ensuring pre-positioned and rapidly deployable antibiotic stocks for outbreak scenarios, aligned with the ambitions of the UK NAP for AMR 2024–2029. Acting with urgency must be matched by strengthening systems to ensure the effective use of antibiotics and to improve long-term outbreak preparedness.

## Supplementary Material

dlag066_Supplementary_Data

## Supplementary data

Supplementary data available as Supplementary data at *JAC-AMR* Online.
