# Supplementary material for: The kent meningococcal outbreak 2026: a wake-up call for antimicrobial stewardship, vaccine policy and outbreak preparedness
Source: JAC Antimicrob Resist. 2026 Apr 27;8(2):dlag066. doi: 10.1093/jacamr/dlag066 (PMC13111483; doi:10.1093/jacamr/dlag066)
Supplement: dlag066_Supplementary_Data [file dlag066_supplementary_data.docx]

**Supplement Figure 1.** Application of the GUIDE Antimicrobial Stewardship Framework to the Kent meningococcal outbreak 2026.


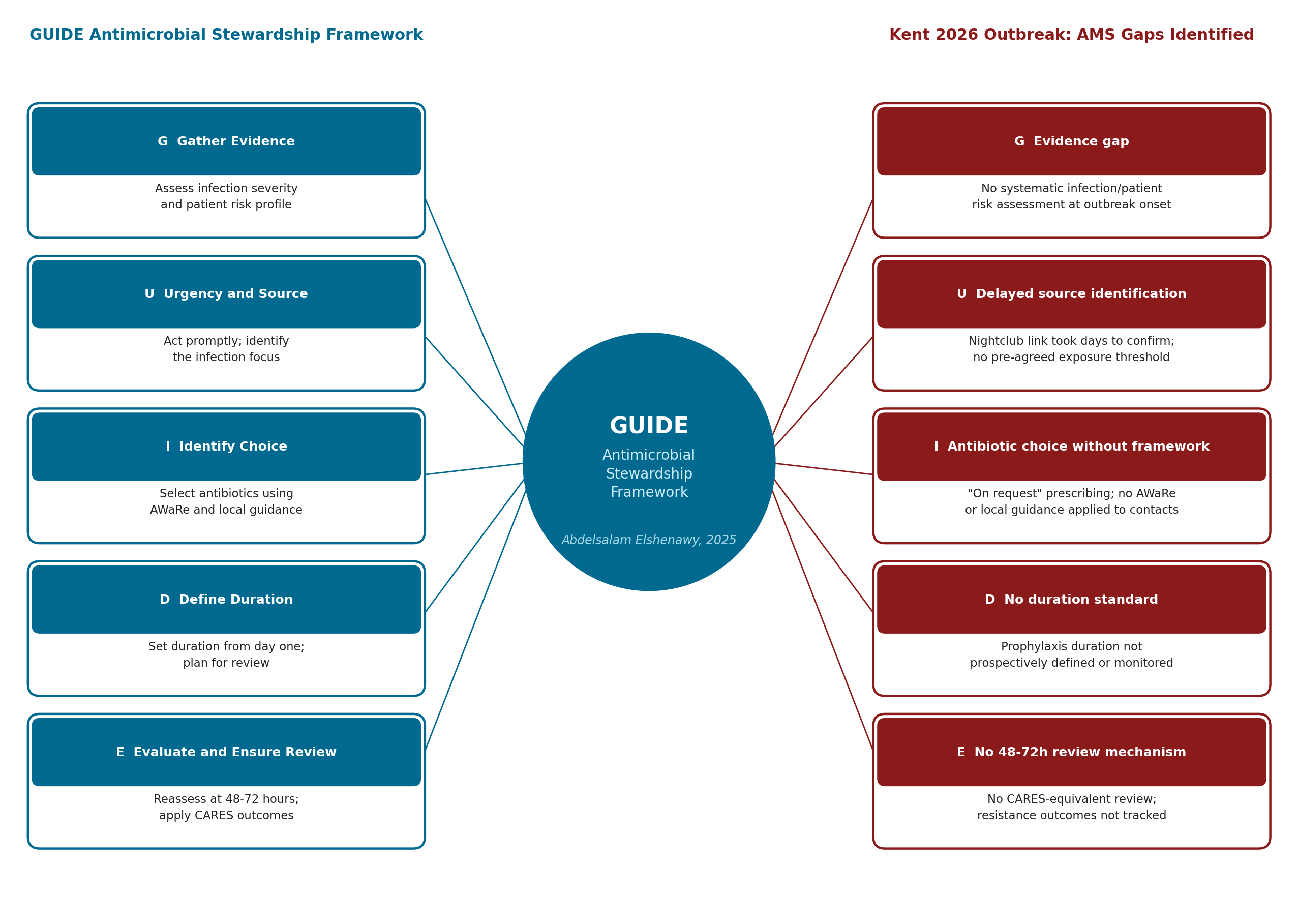


*Supplement Figure 1. Application of the GUIDE Antimicrobial Stewardship Framework (Abdelsalam Elshenawy, 2025) to the Kent meningococcal outbreak 2026, illustrating how each of the five GUIDE steps (Gather Evidence, Urgency and Source, Identify Choice, Define Duration, Evaluate and Ensure Review) maps to a structural AMS gap in the outbreak response. AMS = antimicrobial stewardship; AMR = antimicrobial resistance; AWaRe = Access, Watch, Reserve; CARES = Cease, Amend, Refer, Extend, Switch; UKHSA = UK Health Security Agency.*
